# Signature of microRNA expression during osteogenic differentiation of bone marrow MSCs reveals a putative role of miR-335-5p in osteoarthritis

**DOI:** 10.1186/s12891-015-0652-9

**Published:** 2015-08-05

**Authors:** Pilar Tornero-Esteban, Luis Rodríguez-Rodríguez, Lydia Abásolo, María Tomé, Pedro López-Romero, Eva Herranz, Manuel A. González, Fernando Marco, Enrique Moro, Benjamín Fernández-Gutiérrez, José Ramón Lamas

**Affiliations:** 1 Rheumatology Service, Instituto de Investigación Sanitaria del Hospital Clínico San Carlos (IdISSC). UGC de Reumatología, Hospital Clínico San Carlos, 4a Planta, Ala Norte. C/ Profesor Martín Lagos s/n, 28040 Madrid, Spain; 2 Department of Regenerative Cardiology, Centro Nacional de Investigaciones Cardiovasculares Carlos III, Madrid, Spain; 3 Instituto de Investigación Sanitaria del Hospital Clínico San Carlos (IdISSC). UGC de Traumatología, Hospital Clínico San Carlos, Madrid, Spain

## Abstract

**Background:**

The aim of this study was to evaluate, the existence of a signature of differentially expressed microRNAs (miRNAs) during osteogenic differentiation of bone marrow MSCs from OA and healthy donors and to describe their possible implication in joint regeneration through modulation of molecular mechanisms involved in homeostatic control in OA pathophysiology.

**Methods:**

Following phenotypic assessment of BM-MSCs obtained from OA diagnosed patients (*n* = 10) and non-OA (*n* = 10), total small RNA was isolated after osteogenic induction for 1, 10 and 21 days, miRNA profiles were generated using a commercial expression array of 754 well-characterized miRNAs. MiRNAs, with consistent differential expression were selected for further validation by quantitative reverse-transcription polymerase chain reaction (qRT-PCR) analysis.

**Results:**

A total of 246 miRNAs were differentially expressed (fold change ≥ ± 2, *P* ≤0.05) between OA and non-OA BM-MSC samples; these miRNAs showed variable interactions depending on the cell and differentiation status. Two miRNAs, hsa-miR-210 and hsa-miR-335-5p out of 21 used for validation showed a significant downregulated expression during induced osteogenesis. In particular hsa-miR-335-5p, a critical regulator in bone homeostasis, was further studied. hsa-miR-335-5p downregulation in OA-MSCs, as well as their host coding gene, *MEST*, were also assessed.

**Conclusions:**

To our knowledge, this study represents the most comprehensive assessment to date of miRNA expression profiling in BM-MSCs from OA patients and their role during osteogenic differentiation. We describe the existence of a correlation between miR-335-5p expression and OA indicating the putative role of this miRNA in OA features. These findings, may contribute to our understanding of the molecular mechanisms involved in MSCs mediated homeostatic control in OA pathophysiology that could be applicable in future therapeutic approaches.

**Electronic supplementary material:**

The online version of this article (doi:10.1186/s12891-015-0652-9) contains supplementary material, which is available to authorized users.

## Background

Osteoarthritis (OA) is a common musculoskeletal disorder with high socioeconomic impact [1]. It is characterized by alterations in bone-cartilage homeostasis leading to progressive degeneration of synovial joints [2]. OA etiology is largely unknown; however it has been associated to susceptibility factors such as age, prior joint injury and other alterations linked to genetic and epigenetic factors [3, 4]. Currently, no cure exists for OA, beyond pain relief. In this context, cell therapies are being studied as an alternative for OA treatment [5]. Therapeutic approaches using MSCs (*Mesenchymal Stem* in *Cells*) are considered one of the best options for future treatment of damaged OA cartilage and bone. This approach is based on the potential of MSCs to differentiate into chondrocytes and osteoblasts and their immunomodulatory properties.

Biochemical mechanisms involved in OA initiation and progression are modulated by a fine-tuned crosstalk between different signaling pathways, most of them converging in the WNT signaling pathway [6]. WNT signaling is involved into a range of biological processes essential for maintenance of joint homeostasis, including cell proliferation, cell-fate determination and differentiation [7–9]. In addition to cell signaling, another important regulatory mechanism of cell function is the post-transcriptional modulation of gene expression by microRNAs (miRNAs), a family of small noncoding RNAs. MiRNAs have been described as important players interfering in the expression of multiple target genes and thus modulating their biological functions, e.g. chondrogenesis and osteogenesis [10–12].

Dysfunction of canonical Wnt/β-catenin signaling has been implicated in OA pathogenesis [13]. A number of components including Wnts, frizzled, secreted frizzled-related protein (sFRP), Dickkopf and LRPs (LDL-receptor-related protein) play crucial roles during cartilage and bone development and joint maintenance. Moreover, increased levels of β-catenin have been observed in degenerative cartilage, suggesting that continuous Wnt signaling might contribute to cartilage loss. This evidence was supported by the fact that a polymorphism in the *SFRP3* is associated with a reduced ability to limit β-catenin signaling and with an increased susceptibility to OA development [14].

Together, these evidences suggest that deregulation of signaling pathways by miRNAs and their relationship may provide clues to understand the OA pathogenesis. Indeed, Wnt β-catenin signaling pathway has been described to be susceptible of modulation by targeted repression of translation of specific pathway components [15, 16]. For example, increased expression levels of miR-335-5p regulate bone development promoting osteogenic differentiation by downregulating DKK1 and thus activating Wnt signaling [17]. On the other hand, some miRNAs are also specifically expressed under regulation of Wnt/β-catenin in a regulatory feedback loop [18, 19]. Moreover, tissue damage or proinflammatory signals may also cause miR-335 downregulation which in turn activates the proliferative, migratory and differentiation capacities of MSCs [20].

Given that miRNAs are important post-transcriptional regulators of gene expression, variations in miRNA expression levels are likely involved in molecular changes occurring during joint formation and/or remodeling [21]. To describe the role of miRNAs in OA pathophysiology, we aimed to evaluate the existence of a differential expression signature of miRNAs in bone marrow MSCs comparing OA and healthy donors during *in vitro* induced osteogenesis. This knowledge is essential, both for better understand the regulatory mechanisms taking place in OA pathophysiology and for development of new therapeutic approaches based in selective targeting of key molecules.

## Methods

### Patients and specimens

Bone marrow aspirates were obtained from patients undergoing total hip arthroplasty of OA patients (*n* = 10) (71.2 ± 9.2 years) and control subjects (*n* = 10) with femoral neck fracture (82.0 ± 9.5 years). OA diagnosis was established according to the ACR criteria [22]. Control subjects, evaluated by an independent observer, did not show OA radiographic changes and their clinical records did not evidenced signs of osteoporosis assessed by densitometric T-score > −2.5 SD. All samples were processed after written informed consent was obtained. The study was approved by our institutional ethics committee (CEIC Hospital Clínico San Carlos) according to the principles expressed in the Declaration of Helsinki.

### BM-MSCs cell cultures

MSCs were obtained from 5 to 10 ml of bone marrow aspirates. Briefly, each aspirate was diluted 1:1 with Dulbecco’s modified Eagle’s medium (DMEM) (Promocell GmbH, Heilderberg, Germany) and layered over an equal volume of Ficoll. After centrifugation, at 900 × g for 30 min, the mononuclear cell layer was recovered, washed with DMEM, and re-suspended in DMEM supplemented with 10 % fetal bovine serum 100 U/ml penicillin, 100 mg/ml streptomycin, and 2 mM L-glutamine. The cultures were washed to remove the non-adherent cells and further expanded until approximately 80 % confluence. Cells at confluence were detached with 0.25 % trypsin-EDTA for 5 min at 37 °C and replated for continued passaging at dilution 1:2. Cells at the third passage were used for experiments.

### BM-MSCs characterization by flow cytometry and lineage multipotential

Phenotypic characterization of isolated MSCs was performed by flow citometry in a Gallios flow cytometer (Beckman Coulter). Cells recovered at the third passage were washed with PBS prior incubation during 30 min at 4 °C with specific phycoerythrin conjugated antibodies and their isotype matched antibodies (all from Miltenyi Biotech). Expression of CD105, CD73 and CD90, and lack of expression of CD45, CD34, and CD14 were the minimal criteria required prior to evaluation of their differentiation potential towards chondrogenic, adipogenic and osteogenic lineages, assessed by histochemical stainings.

### Induction of osteogenic, adipogenic and chondrogenic differentiation

Cultures for histochemical analysis were performed in 24-well tissue culture plates at a seeding density of (30,000 cells/cm^2^) using commercial culture media with supplements according to manufacturer instructions (Promocell Cat# C-28013 for osteogenesis, Cat# C-28012 for chondrogenesis and Cat# 28011 for adipogenesis).

The degree of mineralization in osteogenic cultures was assessed after staining for 20 min with 2 % Alizarin red S (Sigma). Adipogenic lineage commitment was evaluated in adipogenic induced cultures, by staining for 5 min with an Oil red O working solution (3 parts of 0.5 % Oil Red O in isopropanol diluted with 2 parts of distilled water). Chondrogenic potential was evaluated by measuring proteoglycan synthesis in chondrogenic induced cultures. Staining was performed for 20 min with 1 % Alcian Blue. Visualization of cells was performed under phase contrast microscopy (Leica 4000b DMI, Leica. Germany).

### Extraction of miRNAs and microarray analysis

Total small RNA was recovered at days 1, 10 and 21 of osteogenically induced MSCs. RNA was solated using the mirVana PARIS kit (Ambion, Grand Island, NY, USA) according to the manufacturer’s instructions. MiRNA quality was assessed using Agilent’s 2100 bioanalyzer (Agilent Technologies, Santa Clara, CA, USA). Total miRNA profiles were generated using an ABI Taqman OpenArray MicroRNA pools A and B to measure the expression of 754 well-characterized miRNA sequences from the Sanger miRBase v14.

Briefly, total small RNA isolated from cells was divided equally for each sample and used with TaqMan Megaplex RT primer pools A or B to generate cDNA which was subsequently amplified using the corresponding Megaplex PreAmp Primers (pools A or B, respectively) following the manufacturer’s instructions. Real-time PCR was performed on the Taqman Open Array MicroRNA plates using the Applied Biosystem Open Array Real-Time PCR system. Data were further exported for analysis (included in Additional file 1).

### Total RNA isolation, cDNA synthesis and RT-PCRs

For miRNA RT-PCR, total RNA was isolated with miRCURY isolation kit (Exiqon) and cDNA Synthesis was carried out using 10 ng of RNA and the Universal cDNA synthesis kit II (Exiqon). Following reverse transcription, cDNAs were 1:100 diluted and loaded onto the real time PCR plates using a 1:1 mixture of Exilent SYBR Green master mix 2× (Exiqon). Real Time PCR reactions were performed in a 7900HT Fast Real time PCR System (Applied Biosystems) under the following conditions: 95 °C 10 min, 45 cycles of 95 °C 10s and 60 °C 1 min. After run completion, Ct values were calculated using the SDS 2.4 software (Applied Biosystems) and data were analyzed using the StatMiner® software (Integromics). The data sets supporting the results of this article are available in the GEO repository, http://www.ncbi.nlm.nih.gov/geo/query/acc.cgi?acc=GSE70856.

Total RNA was isolated from cultured cells with a miRNeasy Mini Kit (Qiagen, Valencia, CA, USA). Human miR-335 and *MEST*, were quantified by real-time RT-PCR using the corresponding TaqMan Gene Expression Assays (Applied Biosystems, Foster City, CA, USA). *RNU6B* and *GAPDH* were used as endogenous normalization controls for miRNAs and *MEST*, respectively.

For PCR amplification of *DKK1* and *SFRP1*, RNA from cultures was pre-amplified using the RT^2^ Nano PreAMP cDNA Synthesis Kit (Cat# C06, SABiosciences, Qiagen).

cDNA conversion was performed with the Maxima H Minus Kit (Cat#K1682. Fermentas) and the Maxima Sybr/R QPCR kit (Cat# K0221. Fermentas) was used for PCR amplification in a Mastercycler realplex4 (Eppendorf) using the following conditions: 95 °C, 10 min; 40 cycles of (95 °C, 15 s; and 60 °C, 60 s). Forward and Reverse primer sequences used were respectively: *ACTB* (β-actin), 5′-CGC CCC AGG CAC CAG GCG-3′; 5′-GCT GGG GTG TTG AAG GT-3′. *DKK1*: 5′-GTG CAA ATC TGT CTC GCC TG-3′; 5′-GCA CAG TCT GAT GAC CGG AG-3′ and *SFRP1*: 5′-TTT GAG GAG AGC ACC CTA GGC-3′; 5′-TGT GTA TCT GCT GGC AAC AGG-3′. *ACTB* was used as internal reference gene. Amplification of the relevant amplicon sizes (284, 266 and 75 bp respectively) was confirmed by electrophoresis on a 2 % agarose gel stained with Midori Green (Nippon Genetics Europe GmbH) and visualized under ultraviolet light.

### Data analysis and statistical methods

Microarray data preprocessing was done with the R programming language. MiRNAs with Ct values higher than 35 were considered as undetected. Data were normalized using a mean-centering restricted (MCR) strategy as described by Wylie et al. [23], which uses miRNAs expressed in all samples for data normalization. The mean of these fully expressed miRNAs in a given sample is subtracted from all miRNAs in that sample. After normalization, statistical analysis was performed via custom scripts based on the R/Bioconductor package LIMMA (Linear Models for Microarray) [24]. Comparisons between experimental groups were performed using a moderated *t*-test from LIMMA and *P*-values were adjusted for multiple testing using the Benjamini-Hochberg method [25]. Adjusted *P*-values < 0.05 were considered statistically significant. Unless specified, median and interquartile rank (IQR) values were calculated. *P* values less than 0.05 were considered significant. Data were analyzed using Graph-Pad Prism 6.01 (GraphPad Software, San Diego, CA).

## Results

### Characterization of bone marrow MSCs

**Fig. 1:**
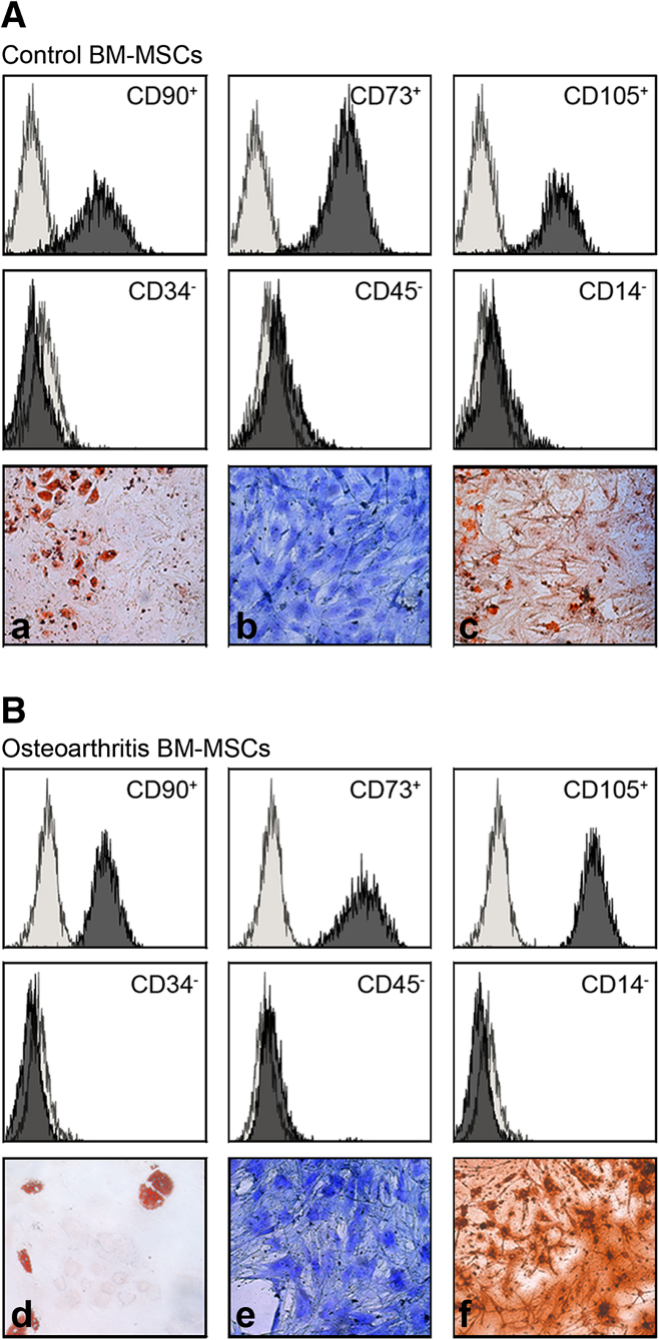
Characterization of BM-MSCs by Flow Citometry and *in vitro* potential of differentiation. Representative flow Cytometry analysis of bone marrow MSCs obtained from one Control (non-OA) donor (**a**). and one osteoarthritic patient (**b**). MSCs at third passage were positive for MSC specific markers (CD90, CD73, CD105 and negative for CD34, CD45 and CD14). Figure represents an overlay image of antibody isotype controls (light gray histogram) and specific marker antibodies (dark gray histogram). Histochemical staining of *In vitro* potential of differentiation through the adipogenic, chondrogenic and osteogenic lineages measured at day 21 (adipogenesis and osteogenesis) or 28 (chondrogenesis) for Control BM-MSCs (a, b and c respectively) and OA BM-MSCs (d, e and f). Magnification × 100

The phenotypic uniformity of BM-MSCs used in the study was assessed according to several minimal criteria: these include the plastic-adherence in culture, positivity for expression of CD90, CD73, CD105 and absence of hematopoietic surface markers CD34, CD45 and CD14 expression and their osteogenic and chondrogenic lineage potential. The expression pattern of the cells used was similar in all samples (Fig. 1a and b) In addition, the multilineage differentiation potential of MSCs used in the study was evaluated. All samples were able to differentiate into osteogenic, chondrogenic and adipogenic lineages under the appropriate stimulatory conditions (Fig. 1a and b).

### MiRNA expression profiles during osteogenic induction using microarray

To address the hypothesis that OA- or Control-MSCs have a different epigenetic signature of miRNAs during osteogenic differentiation. Expression levels of 754 miRNAs were assessed using miRNA microarray expression chips. Raw data were preprocessed and filtered using R routines. A total of 246 miRNAs with at least two observations for each combination of status (OA or Control) and differentiation days (t = 0, 10, 21) were further processed. The variability introduced by single individuals was detected using statistical random effects models. Given that no significant effect was detected, data were finally analyzed using fixed effect models.

Combinations of variables which best explained the data were detected after a linear discriminant analysis (LDA). Four miRNAs; hsa-miR-197, hsa-miR-320, hsa-miR-616 and hsa-miR-99b_ showed differences during differentiation indicating the existence of an interaction between status and differentiation variables (Additional file 2). On the other hand, 15 miRNAs did not show any interaction (that is, were independent of cell status) but showed differences during osteogenic progression (Additional file 3). Similarly, 30 miRNAs were independent during differentiation times analyzed but showed differences between OA and control samples (Additional file 4). However, although all the differences noted were statistically significant after multiple test correction this significance was lost. The data set supporting these results are included in Additional file 1.

### MiRNA validation by quantitative PCR

A set of miRNAs with consistent differential expression in our microarray was further validated by q-PCR. This included a total of 21 miRNAs selected according to their lower q values (Table 1). Distribution of miRNAs analyzed and their overlapping among different comparisons are provided in Additional file 5.

**Table 1:** miRNAs with consistent differential expression in the microarray and their target sequences, validated by q-PCR. This included a total of 21 miRNAs selected according to their lower q values

microRNA name	Target sequence
*hsa-miR-103a-3p*	AGCAGCAUUGUACAGGGCUAUGA
*hsa-miR-134*	UGUGACUGGUUGACCAGAGGGG
*hsa-miR-181a-5p*	AACAUUCAACGCUGUCGGUGAGU
*hsa-miR-191-5p*	CAACGGAAUCCCAAAAGCAGCUG
*hsa-miR-193b-3p*	AACUGGCCCUCAAAGUCCCGCU
*hsa-miR-197-3p*	UUCACCACCUUCUCCACCCAGC
*hsa-miR-210*	CUGUGCGUGUGACAGCGGCUGA
*hsa-miR-222-3p*	AGCUACAUCUGGCUACUGGGU
*hsa-miR-222-5p*	CUCAGUAGCCAGUGUAGAUCCU
*hsa-miR-24-2-5p*	UGCCUACUGAGCUGAAACACAG
*hsa-miR-27b-3p*	UUCACAGUGGCUAAGUUCUGC
*hsa-miR-296-5p*	AGGGCCCCCCCUCAAUCCUGU
*hsa-miR-30c-5p*	UGUAAACAUCCUACACUCUCAGC
*hsa-miR-335-5p*	UCAAGAGCAAUAACGAAAAAUGU
*hsa-miR-370*	GCCUGCUGGGGUGGAACCUGGU
*hsa-miR-379-3p*	UAUGUAACAUGGUCCACUAACU
*hsa-miR-410*	AAUAUAACACAGAUGGCCUGU
*hsa-miR-433*	AUCAUGAUGGGCUCCUCGGUGU
*hsa-miR-497-5p*	CAGCAGCACACUGUGGUUUGU
*hsa-miR-539-5p*	GGAGAAAUUAUCCUUGGUGUGU
*hsa-miR-543*	AAACAUUCGCGGUGCACUUCUU
*hsa-miR-628-3p*	UCUAGUAAGAGUGGCAGUCGA
*hsa-miR-628-5p*	AUGCUGACAUAUUUACUAGAGG

**Fig. 2:**
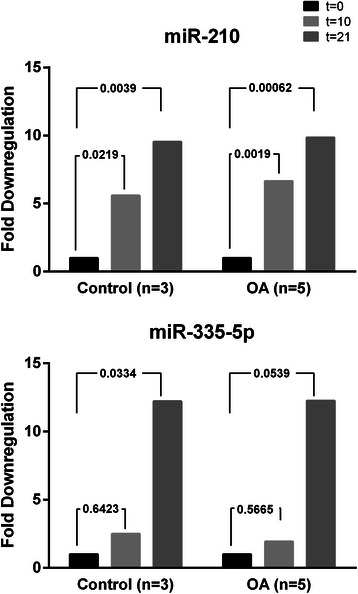
Relative Quantification of comparisons between t = 10 and t = 0; t = 21 and t = 0 of significant miRNAs in OA-MSCs and Control-MSCs. Expression of miR210 and miR-335-5p follow a similar downregulation trend during osteogenic differentiation of MSCs, both in OA (*n* = 5) or in Control subjects (*n* = 3). Project was normalised using: hsa-miR-103a-3p; hsa-miR-191-5p; hsa-miR-30c-5p; SNORD49A. Test Type used: Parametric test (Limma). FDR method: Benjamini-Hochberg Adjusted *p*-value threshold: 0.05

Two miRNAs, hsa-miR-210 and hsa-miR-335-5p out of the 21 used for validation showed a significative downregulated expression during osteogenesis. More specifically, hsa-miR-210 expression pattern was quite similar in OA and control MSCs, which is reflected by an average downregulation of 5.5 times between days [0–10] and 9.5 times between days [0–21]. A downregulatory trend also occurs during osteogenesis for miR-335-5p, which was down-regulated an average of 2 and 12 fold respectively, although not significant in the case of OA-MSCs (Fig. 2).

### BM-MSCs from OA patients express lower levels of miR-335 than control subjects

Current findings have described miR-335 as a critical regulator in bone homeostasis and MSCs commitment. Besides, it has also been described that expression levels of mature miR-335 in human MSCs correlate with those of its host coding-gene *MEST* (mesoderm-specific transcript homolog). Therefore, we aimed to determine whether expression of miR-335-5p correlated with OA status measuring the expression of miR-335-5p and *MEST* in undifferentiated BM-MSC populations from OA and control subjects.

**Fig. 3:**
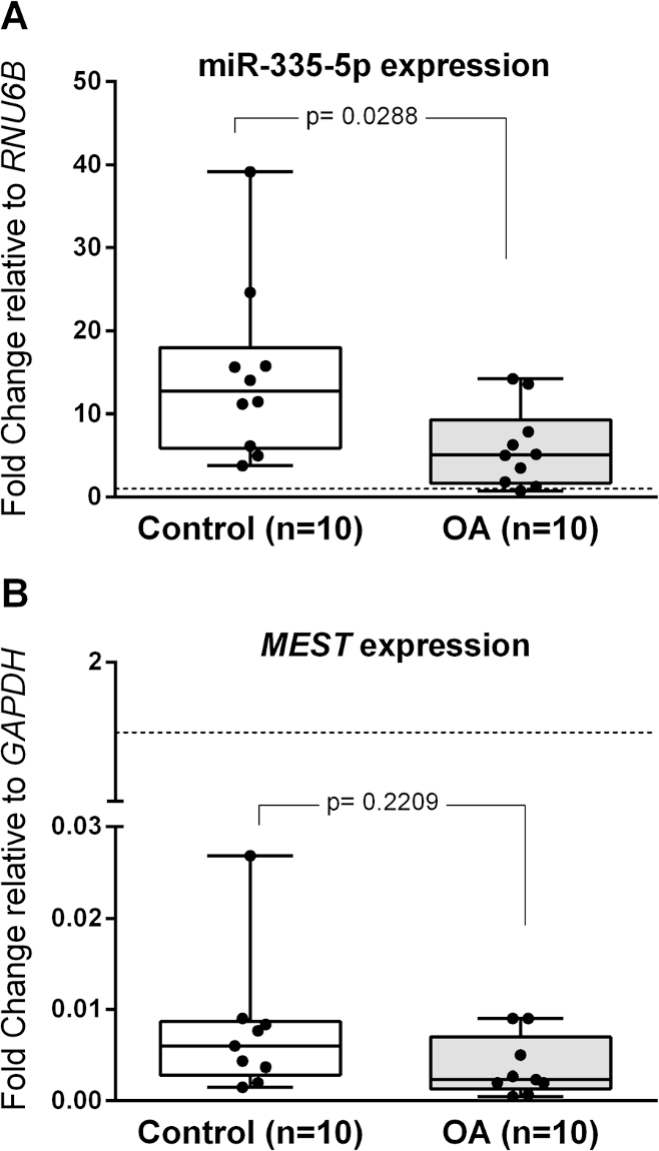
miR-335-5p and *MEST* expression in OA-MSCs and Control-MSCs. **a**. Box and wiskers plot of miR-335-5p expression in OA-MSCs and Control-MSCs relative to the *RNU6B* internal control gene. **b**. *MEST* expression in human in OA-MSCs and Control-MSCs relative to the *GAPDH* reference gene. A positive correlation between miR-335-5p and *MEST* expression was detected (Spearman correlation coefficient, *r* = 0.59; *p* = 0.0961 in OA and *r* = 0.6; *p* = 0.0968 in controls

Our results showed lower expression levels of miR-335 in BM-MSCs obtained from OA donors (Fig. 3a). Additionally, although a positive correlation between miR-335-5p and *MEST* expression was detected (Spearman correlation coefficient, *r* = 0.59; *p* = 0.0961 in OA and *r* = 0.6; *p* = 0.0968 in controls) *MEST* expression was similar between OA and control MSCs (Fig. 3b). These results clearly indicated that downregulation of miR-335-5p was an inherent feature of OA-MSCs.

### Wnt antagonists DKK1 and SFRP1 are not differentially inhibited by miR-335-5p

**Fig. 4:**
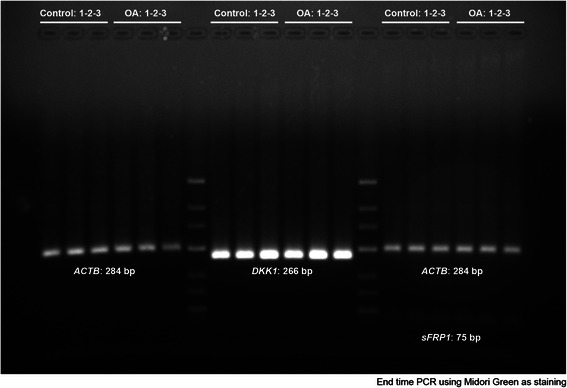
Two percent agarose gel of *DKK1* and *SFRP* amplification products from OA-MSCs and Control-MSCs. After synthesizing the first strand cDNA, it was amplified using PCR. The PCR product was analyzed by agarose gel electrophoresis. PCR reactions (25ul) were loaded as follow: Lanes [1–6] *ACTB* [8–13] *DKK1*. [15–20] ACTB + SFRP1. Lanes 7 and 14 correspond to the molecular markers (GeneRuler^™^ Low Range DNA Ladder, Fermentas). This result shows that single specific PCR product was obtained with expected molecular amplicon sizes. No differences were found in band intensity between OA-MSCs and Control-MSCs

Increased bone formation is one of the main OA features. In addition osteoinduction through Wnt signaling can be promoted by inhibition of Wnt antagonists such as DKK1 and SFRP1, two known predicted targets of miR-335-5p. We next aimed to confirm if differential expression of miR-335-5p in OA-MSCs correlated with a reduced expression of: *DKK1* and *SFRP1.* Our data revealed that independently of miR-335-5p expression, mRNA levels of antagonists were similar both in undifferentiated OA- or Control-MSCs (Fig. 4).

## Discussion

Bone homeostasis, which largely depends on the appropriate differentiation of chondro- and osteo-progenitor MSCs, is susceptible of disturbances leading to skeletal dysplasias or articular diseases such as osteoarthritis [26], however it has been described that during OA progression bone marrow OA-MSCs exhibit different properties that from healthy age-matched MSCs [27]. These alterations are characterized by a lower proliferative capacity, lower adipogenesis and chondrogenesis and increased osteogenesis in OA-MSCs. These changes in OA-MSCs can be attenuated or reversed by particular supplementation of culture media [28]. This could explain why in our *in vitro* cultures cell proliferation and differentiation was quite similar.

Although the role of MSCs still remains unknown, and the exact pathogenetic mechanisms of OA are unravelled, the central role of WNT signaling pathway is considered essential [6]. The Wnt signalling participates during skeletogenesis and regulation of bone remodelling processes in adult tissues through the expression of target genes during endochondral ossification [29]. However, the role of Wnt signalling in osteogenesis is by far controversial. While some authors have reported that its activation promotes osteogenesis [30, 31], others argued the detrimental effects in osteogenesis upon Wnt activation [32, 33]. Moreover, Liu et al. [34] recently described that Wnt promoted the osteogenesis in normal medium while inhibited it the osteogenic differentiation medium. This dual effect was attributed to a regulatory loop between miR-17 and its target (*TCF3*). Taken together, these contradictory results suggest the critical effect of experimental conditions or microenvironmental and epigenetic factors rather than genetic differences. In this regard, a recent report described that reduced methylation in the osteoarthritic bone, compared to osteoporotic bone, results in an upregulated expression of genes and increased WNT pathway activity [35].

As occurs with other signalling pathways, regulation takes place at different levels. Post-transcriptional regulation by miRNAs have emerged as key modulators of signal transmission and cellular response to extracellular signals [36]. In this study we performed a comparative profiling of miRNA expression during *in vitro* osteogenic differentiation of BM-MSCs isolated from OA and Control subjects. We aimed to describe a signature of miRNAs potentially involved in the deregulated osteogenesis occurring in OA.

The role of miR-335-5p on Wnt signalling has been described primarily in cancer. Its implication in bone development has been only partially evidenced. At least 30 genes, related or belonging to the Wnt pathway, have been previously described with varying degrees of evidence as validated targets of this miRNA [17, 37–40] (Table 2). In our study, after filtering of 754 initially screened miRNAs and further validation of 21 of them, only hsa-miR-210 and hsa-miR-335-5p showed significative differences in expression during osteogenesis. Subsequently, using the latest miRTarBase database, we analyzed if these miRNAs showed experimentally validated miRNA-target interactions in the Wnt pathway. Given that only miR-335-5p showed the existence of direct potential targets we focused our attention on this miRNA. (Additional file 6).

**Table 2:** Genes implicated in WNT pathway, matrix and cytoskeleton described as targets of hsa-miR-335-5p

WNT related genes			Other		
Gene	EntrezID	Reference	Gene	EntrezID	Reference
*AXIN1*	8312	39	*RUNX2*	860	20
*CCND2*	894	39	*COL1A1*	1277	40
*CTNNBIP1*	56998	39	*COL10A1*	1300	39
*DAAM1*	23002	39	*COL11A1*	1301	39
*DKK1*	22943	17	*COL15A1*	1306	39
*DKK2*	27123	39	*COL21A1*	81578	39
*DKK4*	27121	39	*COL3A1*	1281	39
*FGF4*	2249	39	*COL4A5*	1287	39
*FZD1*	8321	39	*COL6A1*	1291	39
*FZD10*	11211	39	*COL6A5*	256076	39
*FZD8*	8325	39	*COL6A6*	131873	39
*LRP5*	4041	39	*COL8A2*	1296	39
*LRP6*	4040	39	*COL9A2*	1298	39
*MYC*	4609	37, 39	*COL9A3*	1299	39
*NFAT5*	10725	39			
*PPP1R1A*	5502	39			
*PPP2R5A*	6422	39			
*PPP3R2*	5528	39			
*PRICKLE2*	166336	39			
*PRKCG*	5582	39			
*SFRP1*	6422	39			
*SMAD3*	4088	39			
*TBL1X*	6907	39			
*VANGL2*	57216	39			
*WIF1*	11197	39			
*WNT1*	7471	39			
*WNT10B*	7480	39			
*WNT3*	7473	39			
*WNT7B*	7477	39			
*WNT9A*	7483	39			

In the present study, we have demonstrated the existence of a downregulation of miR-335-5p in OA-MSCs compared to Control-MSCs. Moreover, we demonstrated the existence of a similar and progressive downregulatory trend of this miRNA along the osteogenic differentiation Control-MSCs not significant in the case of OA-MSCs. Decreased expression of Wnt antagonists such as DKK1 and SFRP1 are likely a major cause of increased Wnt/β-catenin signalling. In this particular, expression of miR-335-5p has been previously described as a key regulator of bone development promoting the downregulation of DKK1 at initial stages of osteogenesis [17]. This modulatory effect significantly persist at later stages of osteogenesis in Control-MSCs, likely to prevent excessive mineralization by promoting elevated levels of DKK1 and/or SFRP1 through dowregulation of miR-335-5p. Although post-transcriptional regulation by miRNAs usually results in gene silencing by reduction of translation and mRNA levels, we found that miR-335-5p did not exert this inhibitory effect on *DKK1* or *SFRP1* mRNA levels. This result, however, is consistent with evidences suggesting that under particular conditions, such as stress, inhibition of translation initiation and mRNA stabilization can occur simultaneously [41].

Previous observations of Tomé et al. [20] proposed the key regulatory role of miR-335 in MSCs biology, concluding that upregulation of miR-335 impairs the MSC reparative phenotype. Here we provide new insights about the putative role of miR-335 in Wnt signalling throughout osteogenesis in OA pathology suggesting a more active Wnt signalling and thus increased osteogenesis and mineralization in OA. In addition to the modulatory role of WNT pathway by miR-335-5p, several other genes involved in extracellular matrix formation have been also described as targets of this miRNA, in particular collagen genes. Some of them, previously described by our group, were clearly downregulated in OA-MSCs. In particular *COL10A1* [42].

## Conclusions

Collectively, these evidences indicate the existence of a correlation between miR-335-5p expression and OA indicating a putative role of this miRNA. These features are of particular interest given that subchondral and periarticular bone remodelling as well as establishment of a proper subchondral cartilage plays a role in the progression of osteoarthritis (OA). These data contribute to our understanding of the molecular mechanisms involved in MSCs mediating homeostatic control in OA pathophysiology.

## Additional files

Additional file 1: **Data analysis of the expression profile of miRNA sequences, from the Sanger miRBase v14 using the Taqman OpenArray MicroRNA pools A and B.** (XLS 2463 kb) Additional file 2: **miRNAs showing interactions between status (OA and Control) and differentiation time points (*****p*** **values <0.05 without multiple test correction applied.** (TIFF 82 kb) Additional file 3: **miRNAs without status and differentiation interaction that show differential expression between osteogenic differentiation time points (*****p*** **value <0.05 without multiple test correction applied).** (TIFF 123 kb) Additional file 4: **miRNAs without status and differentiation interaction that show differential expression between OA and Control samples (*****p*** **value <0.05 without multiple test correction applied).** (TIFF 157 kb) Additional file 5: **Venn diagrams showing the distribution of miRNAs analyzed and their overlapping among different status and osteogenic time points.** (PDF 19 kb) Additional file 6: **Extraction of all the experimentally validated microRNA-target interactions for hsa-miR-210 and hsa-miR-335-5p from the miRTarBase database. An extraction of miR-335 target genes related to the WNT pathway was also included.** These belong to the Wnt signaling pathway, according to the enriched annotations provided in the Kyoto Encyclopedia of Genes and Genomes (KEGG) 04310. (XLS 2121 kb)

## Abbreviations

BM-MSCs: Bone marrow mesenchymal stem cells (hereinafter referred as to MSCs)
OA-MSCs: Bone marrow mesenchymal stem cells from osteoarthritis patients. hsa-miR-335-5p (hereinafter referred as to miR-335-5p)
