# Supplementary material for: Signature of microRNA expression during osteogenic differentiation of bone marrow MSCs reveals a putative role of miR-335-5p in osteoarthritis
Source: BMC Musculoskelet Disord. 2015 Aug 5;16:182. doi: 10.1186/s12891-015-0652-9 (PMC4526194; doi:10.1186/s12891-015-0652-9)

**List 1:** Control *vs* OA; **List 2**: Globally t=(0-21); **List 3:** Differential miRNAs in Osteoarthritis t= (0-21)				
**Common**	**Unique in List 1**	**Unique in List 2**	**Unique in List 3**	**List 2- List 3**
hsa-miR-210	hsa-miR-181a-5p<br>hsa-miR-193b-3p	hsa-miR-27b-3p	hsa-miR-539-5p<br>hsa-miR-628-5p<br>hsa-miR-433<br>hsa-miR-138-5p<br>hsa-miR-370	hsa-miR-197-3p<br>hsa-miR-222-3p<br>hsa-miR-296-5p<br>hsa-miR-335-5p<br>hsa-miR-628-3p<br>hsa-miR-379-3p

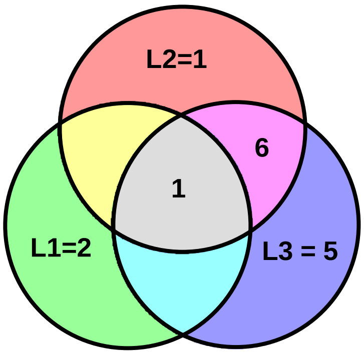

**List 1:** Control *vs* OA; **List 2**: Globally t=(10-21); **List 3:** Differential miRNAs in Osteoarthritis MSCs t= (10-21)				
**Common**	**Unique in List 1**	**Unique in List 2**	**Unique in List 3**	**List 2- List 3**
		hsa-miR-296-5p		hsa-miR-222<br>hsa-miR-24-2-5p<br>hsa-miR-27b-3p<br>hsa-miR-335-5p<br>hsa-miR-370<br>hsa-miR-410<br>hsa-miR-543

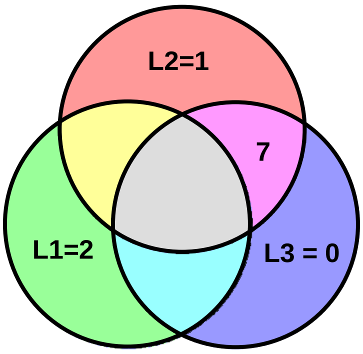

Differential miRNAs between days 0-21 (List 1) and 10-21 (List 2) in MSCs (Globally)

**Common**	**Unique in List 1**	**Unique in List 2**
hsa-miR-222-3p<br>hsa-miR-27b-3p<br>hsa-miR-296-5p<br>hsa-miR-335-5p	hsa-miR-197-3p<br>hsa-miR-210<br>hsa-miR-497-5p<br>hsa-miR-628-3p	hsa-miR-24-2-5p<br>hsa-miR-370<br>hsa-miR-410<br>hsa-miR-543<br>hsa-miR-134

Differential miRNAs between days 0-21 (List 1) and 10-21 (List 2) in Osteoarthritis MSCs

**Common**	**Unique in List 1**	**Unique in List 2**
hsa-miR-222<br>hsa-miR-335-5p<br>hsa-miR-370<br>hsa-miR-433<br>hsa-miR-379-3p	hsa-miR-197-3p<br>hsa-miR-210<br>hsa-miR-296-5p<br>hsa-miR-497-5p<br>hsa-miR-539-5p<br>hsa-miR-628-3p<br>hsa-miR-628-5p	hsa-miR-24-2-5p<br>hsa-miR-27b-3p<br>hsa-miR-410<br>hsa-miR-543<br>hsa-miR-134

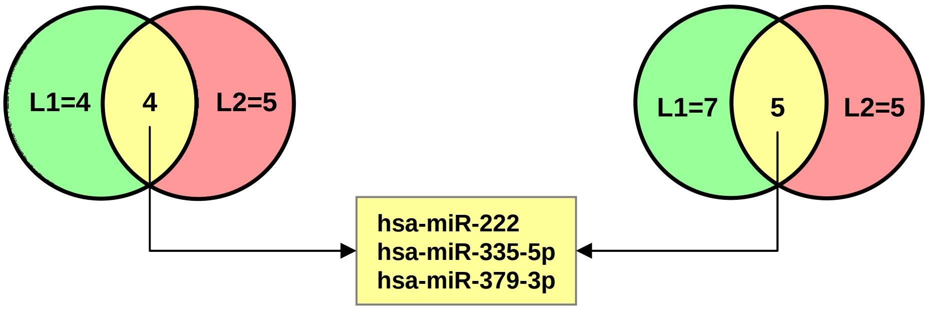

Supplement: Additional file 5: — Venn diagrams showing the distribution of miRNAs analyzed and their overlapping among different status and osteogenic time points. (PDF 19 kb) [file 12891_2015_652_MOESM5_ESM.pdf]
